# m^6^A modification: a new avenue for anti-cancer therapy

**DOI:** 10.1093/lifemedi/lnad008

**Published:** 2023-04-08

**Authors:** Yongtai Bai, Kai Li, Jinying Peng, Chengqi Yi

**Affiliations:** State Key Laboratory of Protein and Plant Gene Research, School of Life Sciences, Peking University, Beijing 100871, China; State Key Laboratory of Protein and Plant Gene Research, School of Life Sciences, Peking University, Beijing 100871, China; Academy for Advanced Interdisciplinary Studies, Peking University, Beijing 100871, China; Peking-Tsinghua Center for Life Sciences, Peking University, Beijing 100871, China; State Key Laboratory of Protein and Plant Gene Research, School of Life Sciences, Peking University, Beijing 100871, China; State Key Laboratory of Protein and Plant Gene Research, School of Life Sciences, Peking University, Beijing 100871, China; Peking-Tsinghua Center for Life Sciences, Peking University, Beijing 100871, China; Department of Chemical Biology and Synthetic and Functional Biomolecules Center, College of Chemistry and Molecular Engineering, Peking University, Beijing 100871, China

**Keywords:** m^6^A, RNA modification, METTL3, FTO, cancer therapy

## Abstract

To date, over 170 different kinds of chemical modifications on RNAs have been identified, some of which are involved in multiple aspects of RNA fate, ranging from RNA processing, nuclear export, translation, and RNA decay. m^6^A, also known as *N*^6^-methyladenosine, is a prominent internal RNA modification that is catalyzed primarily by the METTL3-METTL14-WTAP methyltransferase complex in higher eukaryotic mRNA and long noncoding RNA (lncRNA). In recent years, abnormal m^6^A modification has been linked to the occurrence, development, progression, and prognosis of the majority of cancers. In this review, we provide an update on the most recent m^6^A modification discoveries as well as the critical roles of m^6^A modification in cancer development and progression. We summarize the mechanisms of m^6^A involvement in cancer and list potential cancer therapy inhibitors that target m^6^A regulators such as “writer” METTL3 and “eraser” FTO.

## Introduction

**Figure 1.**
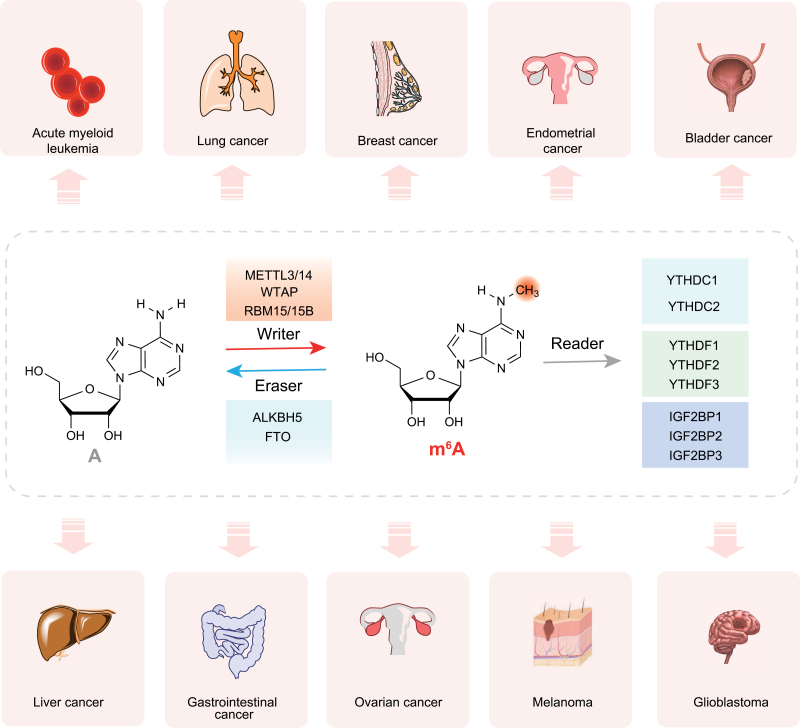
**m** ^**6**^ **A modifications and m** ^**6**^ **A in various cancers.** m^6^A is a dynamic and reversible process coordinated by a series of methyltransferases (“writers”: METTL3/14, WTAP, RBM15/15B), demethylases (“erasers”: FTO and ALKBH5) and identifiers (“readers”: YTHDC1/2, YTHDF1/2/3). We highlight the recent reports and related mechanisms of those m^6^A regulatory proteins in acute myeloid leukemia, breast cancer, lung cancer, endometrial cancer, bladder cancer, liver cancer, gastrointestinal cancer, ovarian cancer, melanoma, and glioblastoma.

Different types of chemical modifications on biological macromolecules (proteins, DNA, RNA, sugars, and lipids) are found in higher eukaryotic cells, and these have been identified over the last five decades by using various methods [1]. RNA modification is not only dynamic and reversible but can also be regulated by a wide range of factors, giving rise to the term “epitranscriptome” and gaining popularity in recent years [2–5]. *N*^6^-methyladenosine (m^6^A, methylation of adenosine at position 6) is the most abundant RNA modification in eukaryotic message RNA (mRNA) [6]. Fat mass and obesity-associated protein (FTO) and alkB homologue 5 (ALKBH5) are two different RNA demethylases that function to reverse m^6^A (Fig. 1), which is dynamic and reversible like DNA and histone methylation [3, 4]. m^6^A plays critical roles in various aspects of RNA metabolism including mRNA instability, mRNA translation, nuclear export of mRNA, pre-mRNA splicing, and the high-level structure [1, 7]. And m^6^A has emerged as a key participant in numerous essential biological processes in normal physiology and diseases, including tumorigenesis [8].

This review focuses on the most recent molecular findings of m^6^A modification and the critical roles of m^6^A modification in cancer progression. We will review recent studies that investigate how m^6^A methylation affects RNA fate, gene expression, genome stability, and chromatin architecture. Most importantly, we summarize the key roles of m^6^A regulatory proteins in various types of cancers, as well as the mechanisms of representative potential cancer therapy inhibitors.

## m^6^A writers, erasers, and readers

m^6^A RNA modification was first identified in the 1970s [9, 10]. m^6^A is an abundant chemical modification (present at an average of 1–2 m^6^A methylated sites per 1000 nucleotides in mammalian cells) of RNA catalyzed by the methyltransferase-like 3 (METTL3)–METTL14 stable heterodimer, and other regulatory factors including WTAP, KIAA1429, ZC3H13, and RBM15/RBM15B [11–18]. Here METTL3 has catalytic activity and METTL14 serves as the RNA-binding scaffold that recognizes the transcripts [12]. WTAP, as METTL3–METTL14 complex interacting partner, also can dramatically affect cellular m^6^A deposition [13]. ZCCHC4 and METTL5 are the main methyltransferases responsible for m^6^A modification of 28S and 18S rRNA, respectively [19, 20].

**Figure 2.**
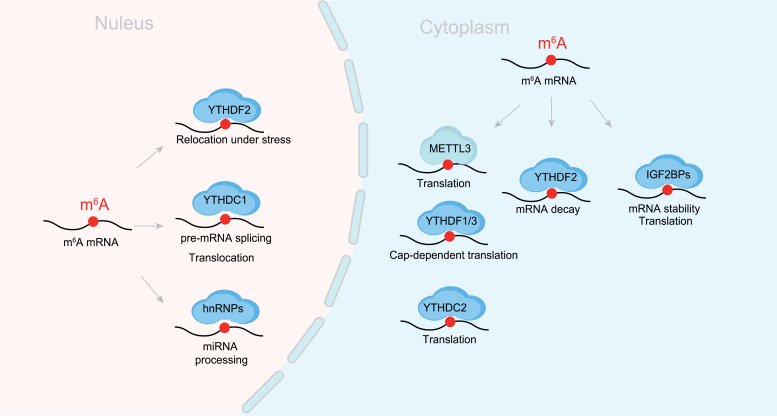
**m** ^**6**^ **A “readers” and their functions.** m^6^A readers mainly characterized contain YTH domain (YTHDC1/2 and YTHDF1/2/3). IGF2BPs and hnRNPs also are m^6^A reader. And those reader proteins are involved in multiple aspects of RNA fate such as RNA splicing and RNA translation.

In 2011, Jia et al. discovered the first m^6^A demethylase: the fat mass and obesity-associated protein (FTO), demonstrating that m^6^A modification is reversible and dynamic [3]. Since then, this field has advanced far too rapidly. Dynamic m^6^A modification has been reported as critical for the development and human diseases, including cancers [21]. In addition to FTO, ALKBH5 was discovered as another mammalian m^6^A demethylase in 2013 [4]. Its demethylation activity significantly affects mRNA export, RNA metabolism, and mouse spermatogenesis [4]. Note that FTO also acts as a demethylase for *N*^6^,2-*O*ʹ-dimethyladenosine (m^6^Am) at the 5ʹ cap and m^1^A in tRNAs, however, ALKBH5 remains the only specific m^6^A demethylase [3, 4]. The m^6^A modifications mostly occur at the DRACH motif (D = G, A, or U; R = G or A; H = A, C, or U), and are enriched in the stop codon vicinity, 5ʹ untranslated region (UTR) and within the coding region [18].

m^6^A readers (Fig. 2) mainly characterized contain YTH domain. There are five YTH domain-containing proteins: YTHDC1, YTHDC2, YTHDF1, YTHDF2, and YTHDF3 [22]. YTHDC1 regulates mRNA splicing by recruiting SRSF3 while blocking SRSF10 mRNA binding [23]. YTHDC2 plays a critical role during spermatogenesis and enhances the translation efficiency of target genes [24]. YTHDF1 is reported to play a vital role in increasing the translation of lysosomal cathepsins in dendritic cells, and the binding of YTHDF2 results in the localization of targeted transcript from the translatable pool to mRNA decay sites for degradation [25, 26]. And YTHDF3 facilitates the translation of its targeted transcript interplay with YTHDF1 and affects the decay of methylated mRNAs through cooperation with YTHDF2 [27, 28]. These findings suggest that all three YTHDF proteins collaborate to influence biological processes related to m^6^A RNA methylation. It’s worth noting that insulin-like growth factor-2 mRNA-binding proteins (IGF2BPs, which include IGF2BP1, IGF2BP2, and IGF2BP3) are also classified as m^6^A-modified mRNA binding proteins [29, 30]. They primarily promote the stability and translation of target mRNAs like the oncogene *MYC* [29]. IGF2BPs have a tandem of two N-terminal RNA recognition motifs (RRM1 and RRM2 domains) and two tandems of two KH domains that are responsible for m^6^A recognition and binding [31].

As a result, m^6^A modifications are written, erased, and read in a highly controlled and context-specific manner (Fig. 1). Furthermore, thanks to recent advances in sequencing and quantification, we can now study m^6^A modifications in the transcriptome in depth. The m^6^A modification is dysregulated in a variety of cancers and is linked to patient prognosis [32]. It has been reported that m^6^A regulators can play oncogenic or tumor suppressor roles in various tumor types [33].

## The role of m^6^A RNA methylation in cancer

### Acute myeloid leukemia

m^6^A “writer” and “eraser” complexes have been characterized earliest in acute myeloid leukemia (AML). The oncogenic roles of METTL3–METTL14 in AML have been demonstrated by accumulating evidence [34–36]. Vu et al. discovered in 2017 that m^6^A modifications play critical roles in regulating the maintenance of human hematopoietic stem/progenitor cells (HSPCs) and myeloid differentiation [34]. METTL3 has been found to be overexpressed in human AML cells compared to healthy HSPCs. METTL3 knockdown also reduced cell proliferation in multiple myeloid leukemia cell lines, along with induced differentiation and apoptosis. Overexpression of a wild-type version of METTL3, but not a catalytically dead mutant, could rescue these phenotypes. Thus, METTL3 enzyme activity is required to maintain these properties [34]. Vu et al. also found METTL3 loss resulted in lower levels of m^6^A on c-MYC, BCL2, and PTEN mRNA, thereby causing a decrease of c-MYC, BCL2, and PTEN protein expressions and possibly activating the PI3K/AKT pathway [34]. Furthermore, Barbieri et al. discovered that the CAATT-box binding protein CEBPZ recruits METTL3 to the promoters of a specific set of active genes, resulting in increased translation [36]. SP1 is a METTL3 target that regulates c-MYC expression and plays oncogenic roles in cancer [37]. It should be noted that METTL3 affects translation in several ways, including (i) being associated with chromatin and localized to the transcriptional start sites of active genes by a METTL14 independent manner; (ii) by promoting RNA loading onto ribosomes; (iii) by recruiting m^6^A reader proteins such as YTHDF1; (iv) interacting with the translation initiation machinery such as eukaryotic initiation factor 3 (eIF3) through an m^6^A-mediated cap-independent manner in 5ʹUTRs [38, 39]. METTL3 may also extend the half-life of ITGA4 mRNA, thereby increasing the expression level of ITGA4 and thus increase AML cells homing/engraftment [35]. Overall, these studies provide detailed mechanisms for the therapeutic targeting of METTL3 in AML.

METTL14, like METTL3, plays an oncogenic role by fine-tuning the expression of its mRNA targets such as *MYB* and *MYC* [40]. METTL14 is downregulated during the process of myeloid differentiation and METTL14 knockdown promotes the differentiation of HSPCs toward myeloid cells, demonstrating that METTL14 plays a critical role in inhibiting normal myelopoiesis [40]. METTL14 depletion resulted in the inhibition of cell growth and apoptosis in AML cells. Additionally, the transcription factor SPI1 was found as a negative regulator of METTL14 in AML cells [40]. It is worth noting that METTL14 depletion had a greater inhibitory effect in leukemia cells than in normal HSPC, implying a potential therapeutic value [40].

Many of the enzymes that regulate protein and DNA modifications are currently targets of cancer therapies; could we target RNA modifications for cancer therapy? Yankova et al. describe METTL3 inhibition as a potential therapeutic strategy against AML in a recent *Nature* report [41]. STM2457, in particular, can directly bind to the METTL3-METTL14 heterodimer and inhibit its catalytic activity [41]. To our delight, STM2457 did not inhibit other RNA methyltransferases or DNA methyltransferases [41]. As far as we know, this means that STM2457 is a first-in-class METTL3 inhibitor.

FTO, but not ALKBH5, has been reported to be significantly upregulated in MLL-rearranged AML compared to normal controls [42]. FTO’s demethylase activity reduces the m^6^A levels of ASB2 and RARA, which in turn downregulates these two genes at the RNA and protein levels [42]. FTO overexpression promotes AML cell proliferation. As a result, FTO plays an oncogenic role in AML [42]. (R)-2-hydroxyglutarate ((R)-2HG) was previously reported as an oncometabolite that interferes with various α-KG- mediated processes, could extend the lifespan of *C. elegans*, and inhibit glioblastoma cancer cell growth and viability [43]. Su et al. found that (R)-2HG has anti-tumor activity via targeting FTO/m^6^A/MYC/CEBPA signaling [44]. They also found (R)-2HG plays important roles in cancer metabolism such as glycolysis via abrogates FTO/m^6^A/YTHDF2-mediated upregulation of PFKP and LDHB [45]. Another promising FTO inhibitor, namely FB23-2, could directly bind to FTO and selectively inhibits FTO’s demethylase activity [46]. Through inhibiting MYC and CEBPA expression and activating p53-mediated apoptosis pathways, FB23-2 significantly inhibits the progression of AML cells *in vitro* and *in vivo* [46]. Those reports suggested that FTO is a druggable target and targeting FTO by small-molecule inhibitors holds the potential to treat AML [46]. Considering FTO is highly expressed on some subtypes of AML cells, it is important to note that FTO inhibitors are effective only in a limited subset of AML patients. In addition, further studies should be investigated whether competitive antagonists of α-ketoglutarate specifically inhibit ALKBH5.

### Breast cancer

METTL3 is highly expressed during breast cancer metastasis, demonstrating that METTL3 plays an oncogenic role in breast cancer and may be a potential therapeutic target [47]. METTL3 promotes breast cancer lung metastasis by enhancing the stability of a keratin 7 (KRT7)-AS/KRT7 mRNA duplex and translation of KRT7 [47]. Interestingly, METTL3 enhances HBXIP expression, HBXIP in turn upregulates METTL3 expression by suppressing let-7g and induces positive feedback of HBXIP/let-7g/METTL3/HBXIP signaling axis to accelerate cell proliferation [48].

Aggressive triple-negative breast cancer is usually considered resistant to chemotherapy with a poor prognosis and regarded as responsive to immunotherapy in recent years. METTL3 functions as an immunomodulator in the breast cancer microenvironment. METTL3 was reported as a potential upstream m^6^A methyltransferase of PD-L1, thereby regulating PD-L1 expression and stability in breast cancer cells [49]. And IGF2BP3 is a reader of PD-L1 m^6^A methylation, which significantly decreases the expression and stability of PD-L1 in breast cancer cells [50]. A deficiency of METTL3 also influences macrophage reprogramming and enhances tumor progression in other tumor mouse models [51]. Taken together, those reports demonstrated that METTL3 may serve as a potential therapeutic target for tumor immunotherapy in the future.

It has been reported that treatment with the chemotherapeutic agent doxorubicin leads to elevate m^6^A modification. Protein arginine methyltransferase 5 (PRMT5) downregulated RNA m^6^A modification under doxorubicin treatment, which enhances ALKBH5’s nuclear translocation [52]. METTL3/miR-221-3p/HIPK2/Che-1 axis as a novel signaling pathway that may be responsible for breast cancer resistance to adriamycin [53]. In addition, ALKBH5 also removed the m^6^A methylation of BRCA1 for mRNA stabilization and timely DNA repair in breast cancer cells [52].

Posttranslational modification of METTL3 also plays a key role in tumor invasion and metastasis. More recently, Li et al. found an acetylation-dependent regulation of METTL3 localization that impacts the metastatic dissemination of breast cancer [54]. In this study, METTL3 acetylation determines METTL3 translocation between the nucleus and cytosol [54]. On the one hand, METTL3 acetylation inhibits the localization of METTL3 to the nucleus, therefore restraining its nuclear functions. On the other hand, the acetyl-mimetic METTL3 K177Q mutant displayed increased translation via METTL3-eIF3h interaction [54]. In response to DNA double-strand breaks (DSBs), METTL3 is activated by ATM-mediated phosphorylation and rapidly localized to DNA damage sites [55]. Phosphorylated METTL3 further recruits YTHDC1 for m^6^A-modified RNA protection. In this way, the METTL3-m^6^A-YTHDC1 axis promotes the accumulation of DNA-RNA hybrids at DSBs for downstream RAD51 and BRCA1 recruitment [55]. Thus, METTL3-deficient cells display defective homologous recombination (HR) and genome instability. Another study also found that ERK phosphorylates METTL3 at S43/S50/S52 and USP5 could deubiquitinate METTL3-METTL14-WTAP m^6^A methyltransferase complex, guaranteeing stabilization of the complex [56]. Lactylation modification at lysine amino acid sites of METTL3 was essential for METTL3 capture target RNA, however, the function of lactylation at non-lysine amino acid sites needs further study [57]. SUMOylation of YTHDF2 significantly enhances its binding affinity of m^6^A-modified mRNAs and subsequently contributes to deregulated gene expressions which bear responsibility for cancer progression [58].

A study also connects the regulation of m^6^A RNA methylation to the specification and maintenance of breast cancer stem cells [59]. The authors demonstrated an important role for ALKBH5 in mediating pluripotency factors such as NANOG expression, which are required for primary tumor formation and metastasis [59].

m^6^A reader proteins were also reported to play critical roles in breast cancer. For example, overexpression of reader protein YTHDF3 correlates with brain metastases in breast cancer patients [60]. Mechanistically, YTHDF3 enhances the translation of m^6^A-enriched transcripts (i.e., ST6GALNAC5, GJA1, and EGFR) associated with brain metastasis [60].

METTL3 was recently identified as a p53-interacting protein that plays an important role in enhancing p53 tumor suppressor activity [61]. The authors proposed that METTL3 inhibition would be most effective in cancers with a p53 pathway defect [61]. Many cancers, including breast cancer, have mutated p53. Therefore, METTL3 inhibitors may be more suitable to tumors with p53 mutations. More researchers are needed to determine the precise mechanisms by which METTL3 promotes or suppresses tumor development.

### Lung cancer

METTL3 was reported to be upregulated in lung adenocarcinoma and play an oncogenic role in promoting cell growth, survival, and invasion [38]. In contrast, METTL14 was not elevated in these patient samples. In this study, METTL3 was found to promote the translation of its targeted transcripts including epidermal growth factor receptor (EGFR) and the Hippo pathway effector TAZ by interaction with the translation initiation machinery [38]. Interestingly, the authors claim that METTL3 promotes translation independent of its catalytic activity and downstream m^6^A reader proteins [38]. Another study published in *Nature* found that loss of METTL3 inhibits tumorigenicity and sensitizes lung cancer cells to BRD4 inhibition [62]. The authors proposed that METTL3 promotes oncogene translation and tumorigenesis through an mRNA looping mechanism and identified METTL3-eIF3h as a potential therapeutic target for lung cancer [62]. Furthermore, Li et al. found mutations of FTO and YTHDF3 were linked to the worse overall survival of lung adenocarcinoma by using bioinformatics [63]. Recently, m^6^A regulators or the so-called m^6^A score could be used for the predictive chemotherapy benefits in small-cell lung cancer patients [64]. It is very interesting and convenient to use m^6^A score to assess early screening and diagnosis of tumors, which requires further efforts for the continuous study.

### Endometrial cancer

In endometrial cancer patients, most of them exhibit reductions in m^6^A methylation that are probably due to either reduced expression of METTL3 or METTL14 R298P mutation [65]. Different from most solid tumors, attenuated m^6^A methylation promotes endometrial cancer cell proliferation, colony formation, migration, and invasion. Mechanistically, reduced m^6^A methylation activates the AKT pathway by downregulating the translation of phosphatase PHLPP2 and the upregulating of transcripts encoding subunits of kinase mTORC2 [65]. Therefore, METTL3 mainly plays a tumor suppressor role in endometrial cancer and there are limited studies on the mechanism so far.

High expression of IGF2BP1 was reported to correlate with a poor prognosis of endometrial cancer. IGF2BP1 recognizes and then stabilizes Paternally Expressed Gene 10 (PEG10) mRNA in the three untranslated region (3UTR), thereby enhancing PEG10 expression, accelerating the cell cycle, and promoting cancer progression [66].

The role of m^6^A in endometrial cancer appears to be distinct from that of other solid tumors, and the reason for this distinction requires further systematic studies.

### Bladder cancer

METTL3 was upregulated in bladder cancer and correlated with poor prognosis of bladder cancer patients, thus METTL3 may serve as an oncogene in this case. Through interacting with the microprocessor protein DGCR8 and positively accelerating pri-miR221/222 processes, METTL3 downregulates PTEN expression and ultimately increased cell proliferation [67]. Consistent with the findings, a study found that loss of METTL3 dramatically repressed cancer cell proliferation, invasion, and xenograft tumor formation via AFF4, NF-κB, and MYC signaling networks [68]. Furthermore, circRNA circ0008399 binds WTAP to promote METTL3-METTL14-WTAP complex formation, increasing the expression of TNF alpha-induced protein 3 (TNFAIP3) by enhancing TNFAIP3 mRNA stability in an m^6^A-dependent manner [69]. Therefore, circ0008399 decreased bladder cancer chemosensitivity to cisplatin, and its high expression was associated with poor prognosis [69]. To summarize, we believe that targeting METTL3 or another m^6^A methyltransferase may be an effective therapeutic strategy for bladder cancer.

### Liver cancer

Expression changes in m^6^A regulators have been verified to result in obvious pathological and physiological aberrations in the liver, including cancer. METTL3 is up-regulated in human hepatocellular carcinoma (HCC) and is associated with poor prognosis in patients with HCC [70, 71]. Specifically, METTL3 represses SOCS2 expression in HCC in an m^6^A-YTHDF2-dependent manner [71]. Additionally, METTL3 was reported to be critical during epithelial-mesenchymal transition (EMT) in HCC [72]. In this study, the authors characterize the potential target involved in m^6^A-regulated EMT of cancer cells, SNAI1, an important transcription factor involved in EMT [72]. EMT induced upregulation of m^6^A in CDS of SNAI1 and promoted YTHDF1-mediated SNAI1 translation [72]. Clinical data further showed that tumor tissues containing higher levels of METTL3, SNAI1, and YTHDF1 correlated with worse prognosis for liver cancer patients [72]. METTL3 down-regulated in human sorafenib-resistant HCC, FOXO3 was identified as a key downstream target of METTL3, and its stability increased through a YTHDF1-dependent manner [73]. Interestingly, HBV was found controls host gene expression to maintain a persistent infection by regulating the host m^6^A modification [74]. Mechanistically, the m^6^A modification of PTEN by HBV regulates innate immunity by inhibiting IRF-3 nuclear transport and may result in virus-associated hepatocarcinogenesis through the destruction of the stability in the PTEN and PI3K-AKT pathway [74].

Hou et al. found that ablated YTHDF2 in human HCC cells or mouse hepatocytes promoted inflammatory response, vascular reconstruction, and cancerous metastatic progression [75]. Mechanistically, YTHDF2 participated in the degradation of m^6^A- dependent interleukin-11 (IL-11) and serpin family E member 2 (SERPINE2) mRNAs [75]. Another study also showed that YTHDF2 promotes the liver cancer stem cell phenotype and cancer metastasis in liver cancer by regulating OCT4 mRNA in an m^6^A-dependent manner [76].

METTL16, a human m^6^A methyltransferase, is also required for the tumorigenesis of hepatocellular carcinoma [77, 78]. Surprisingly, two large-scale CRISPR-Cas9 screening datasets suggest that METTL16 is a more important gene for the survival of various types of human cancer cells than any other METTL gene [77]. We believe METTL16 could be a new target for cancer treatment.

### Gastrointestinal cancer

Many studies have demonstrated that METTL3 promotes cell proliferation and serves an oncogenic role in gastrointestinal cancer by regulating several different pathways such as Wnt/PI3K-AKT signaling pathway [79–81]. And METTL3 protein levels were upregulated in gastrointestinal cancer and associated with poor prognosis [80, 81]. In 2020, Ge et al. reported that the m^6^A levels in peripheral blood RNA were significantly increased in gastric cancer (GC) patients compared with those with benign gastric disease or healthy people [82]. Interestingly, m^6^A levels increased in patients with advanced tumors and decreased in GC patients after surgery [82]. Moreover, increased m^6^A level in the peripheral blood of GC patients was accompanied by the downregulation of demethylases FTO and ALKBH5 [82]. Those results indicate that m^6^A levels in peripheral blood RNA may act as a novel predictive biomarker for GC [82].

In GC, METTL3 could not only facilitate cancer progression via m^6^A-modified RNAs but also bind to numerous non-m^6^A-modified RNAs. Recently, Wei et al. reported that cytoplasmic METTL3 interacted with PABPC1 to promote the translation of a series of oncogenes through an m^6^A-independent mechanism [83].

FTO inhibition could significantly increase the sensitivity of 5-FU in 5-FU-resistant colorectal cancer (CRC) cells via the FTO-SIVA1-YTHDF2 axis [84]. SIVA1 is a key target of the FTO and depletion of it restored 5-FU sensitivity in CRC cells [84]. Depletion of ALKBH5 in lymphocytes specifically induces an expansion of γδT cells by targeting the Jagged1/Notch2 signaling pathway, which confers increased protection against gastrointestinal infection [85].

Predicting the occurrence and development of tumors by using the m^6^A level of RNA in peripheral blood has been a hot topic in recent research and could be a very interesting future direction.

### Ovarian cancer

METTL3 may serve an oncogenic role in the progression of ovarian cancer partially through the PI3K-AKT signaling pathway [86]. Huang et al. showed that FTO is downregulated in ovarian cancer [87]. In contrast to the tumor-promoting function of FTO previously reported in other types of cancers, FTO performs a tumor-suppressive role in ovarian cancer [87]. Moreover, the authors identified two phosphodiesterase genes PDE4B and PDE1C as FTO targets that regulate cAMP signaling and play an important role in maintaining the stemness of ovarian cancer stem cells [87]. FTO and ALKBH5 are downregulated in the PARP inhibitor (PARPi)-resistant ovarian cancer cells compared with parental control, which contributes to PARPi resistance by increasing FZD10 mRNA stability to upregulate the Wnt/β-catenin signaling pathway [88]. YTHDF1 facilitates the translation of EIF3C in an m^6^A-dependent manner and is accompanied by an overall translational level, thereby promoting tumorigenesis and metastasis of ovarian cancer [89]. In ovarian cancer, FBW7 has been shown to inhibit tumor progression by inhibiting YTHDF2-mediated BMF mRNA degradation [90].

Many ovarian cancers have p53 mutations, and it remains to be investigated whether these tumors are more sensitive to METTL3 inhibitors. Furthermore, the discovery of new HR factors in cancer, such as METTL3, may broaden PARPi therapeutic potential.

### Melanoma

Melanoma is a cancer that has a high mortality rate and is easily resistant. FTO has recently been shown to regulate the immune response to melanoma and skin tumorigenesis caused by arsenic and UVB irradiation [91, 92]. In human melanoma, FTO is upregulated, and PD-1 is a potential FTO target gene [91]. Yang et al. found that FTO plays a critical role in anti-PD-1 immunotherapy therapeutic resistance, and that combining FTO knockdown with anti-PD-1 treatment may reduce resistance and improve anti-melanoma response [91]. Intriguingly, another FTO inhibitor, Dac51, has the potential to block FTO-mediated immune evasion while also having synergistic effects with checkpoint blockade, which is a powerful strategy for improving adaptive immune response [92].

It should be noted that in addition to tumors mentioned above (summarized in Fig. 1 and Table 1), m^6^A has been reported in other tumors that will not be discussed in detail in this review. For example, different studies have reported opposing effects on the role of the METTL3-METTL14 complex in glioblastoma [93]. m^6^A also plays additional roles in cancers such as pancreatic cancer and prostate cancer [94–96].

**Table 1.** Summary of the biological function of m^6^A regulators in cancer

Cancer types	Representative m^6^A regulators	Participating molecules/pathways	Oncogene OR tumor suppressor	References
AML	METTL3 METTL14 FTO	c-MYC, BCL2, PTEN, SP1, ITGA4 MYB, MYC, SPI1 ASB2, RAR4, PFKP, LDHB, MYC, CEBPA	Oncogene Oncogene Oncogene	[34-37] [40] [42, 44-46]
Breast cancer	METTL3 ALKBH5	KRT7, HBXIP, let-7g, PD-L1 PRMT5, BRCA1, NANOG	Oncogene Oncogene	[47-49] [52, 59]
Lung cancer	METTL3	EGFR, TAZ, eIF3h p53	Oncogene/Suppressor	[38, 62] [61]
Endometrial cancer	METTL3/METTL14 IGFBP2	AKT pathway PEG10	Suppressor Oncogene	[65] [66]
Bladder cancer	METTL3 WTAP	DGCR8, PTEN, NF-κB, MYC TNPAIP3	Oncogene Oncogene	[67, 68] [69]
Liver cancer	METTL3 YTHDF2 METTL16	SOCS2, SNAI1, PI3K-AKT pathway IL-11, SERPINE2, OCT4 RAB11B-AS1	Oncogene Suppressor Oncogene Oncogene	[70-72] [74] [75, 76] [77, 78]
Gastrointestinal cancer	METTL3 FTO	Wnt/PI3K-AKT pathway, PABPC1 SIVA1	Oncogene Oncogene	[79-82] [84]
Ovarian cancer	METTL3 FTO YTHDF1/YTHDF2	PI3K-AKT pathway FZD10 EIF3C, FBW7	Oncogene Suppressor Oncogene	[86] [87] [89, 90]
Melanoma	FTO	PD-1, NF-κB pathway	Oncogene	[91, 92]
Glioblastoma	METTL3/METTL14	ADAR1	Oncogene Suppressor	[93, 98]

## Perspective

Taken together, m^6^A methylation is essential for cancer cell survival in AML and solid malignancies. Significant progress has been made in our understanding of how dysregulated m^6^A alteration and the associated machinery function biologically in a variety of cancers in recent years. m^6^A regulators can play both an oncogenic and a tumor-suppressive role in many tumors. We must carefully and thoroughly evaluate this situation in light of context-dependent property. Because METTL3 or FTO plays an important oncogenic role in many cancers, considerable efforts have gone into developing small-molecule inhibitors that specifically target METTL3 or FTO for cancer treatment. We believe that METTL3 or FTO inhibitors can significantly inhibit downstream targets expression in tumors with high METTL3 or FTO expression, thereby inhibiting tumor proliferation and metastasis. The damage to normal cells can be greatly reduced by controlling drug dosage. As a result, targeting m^6^A is a promising new therapeutic strategy in cancer. Future research should investigate using m^6^A alone or in combination with other therapies to treat solid tumors. Furthermore, because METTL3 has both methyltransferase activity dependent and independent functions in gene regulation, we should consider both aspects when investigating molecular mechanisms.

The lack of quantitative m^6^A high-throughput detection technology severely limits the studies on the regulation of m^6^A in cancers. At present, m^6^A sequencing technology can only identify modification sites at a low resolution, and cannot achieve single-base quantitative detection of m^6^A. More recently, our group provides a quantitative method called GLORI for transcriptome-wide m^6^A sites [97]. This method uses glyoxal and nitrite-mediated deamination of unmethylated A while keeping modified m^6^A intact [97]. It has the potential to be used to track m^6^A dynamics in a variety of biological contexts, including cancer progression. It will be fascinating to learn more about the underlying mechanisms of m^6^A in cancer. In the future, it will be of interest to determine how each site in a specific transcript contributes to the control of RNA stability and translation, and whether the different modified m^6^A sites on the same transcript have different effects in terms of cancer cell survival.
